# PLK1 Inhibition alleviates transplant-associated obliterative bronchiolitis by suppressing myofibroblast differentiation

**DOI:** 10.18632/aging.103330

**Published:** 2020-06-15

**Authors:** Jizhang Yu, Heng Xu, Jikai Cui, Shanshan Chen, Hao Zhang, Yanqiang Zou, Jing Zhao, Sheng Le, Lang Jiang, Zhang Chen, Hao Liu, Dan Zhang, Jiahong Xia, Jie Wu

**Affiliations:** 1 Department of Cardiovascular Surgery, Union Hospital, Tongji Medical College, Huazhong University of Science and Technology, Wuhan 430030, Hubei, China; 2 Cancer Center, Union Hospital, Tongji Medical College, Huazhong University of Science and Technology, Wuhan 430030, Hubei, China

**Keywords:** chronic allograft dysfunction, myofibroblast differentiation, obliterative bronchiolitis, PLK1, TGF-β1

## Abstract

Chronic allograft dysfunction (CAD) resulting from fibrosis is the major limiting factor for long-term survival of lung transplant patients. Myofibroblasts promote fibrosis in multiple organs, including the lungs. In this study, we identified PLK1 as a promoter of myofibroblast differentiation and investigated the mechanism by which its inhibition alleviates transplant-associated obliterative bronchiolitis (OB) during CAD. High-throughput bioinformatic analyses and experiments using the murine heterotopic tracheal transplantation model revealed that PLK1 is upregulated in grafts undergoing CAD as compared with controls, and that inhibiting PLK1 alleviates OB *in vivo*. Inhibition of PLK1 *in vitro* reduced expression of the specific myofibroblast differentiation marker α-smooth muscle actin (α-SMA) and decreased phosphorylation of both MEK and ERK. Importantly, we observed a similar phenomenon in human primary fibroblasts. Our results thus highlight PLK1 as a promising therapeutic target for alleviating transplant-associated OB through suppression of TGF-β1-mediated myofibroblast differentiation.

## INTRODUCTION

Lung transplantation improves the quality of life of terminal lung disease patients [1]. In spite of advances to prevent acute transplant rejection, chronic allograft dysfunction (CAD) is still a major barrier for long-term survival of patients with transplanted organs [2]. Based on data from the Organ Procurement and Transplantation Network (June 7, 2019), ~10% of lung allografts lose function in the first year, and more than half of the recipients lose their functional allograft within five years. Although immunosuppression treatment can effectively prevent acute rejection, it has little effect on CAD and long-term survival rates [3]. Therefore, new treatment methods and drugs targeting CAD are needed to prolong allograft survival.

Transplant-associated obliterative bronchiolitis (OB), the main cause of long-term morbidity and mortality after lung transplantation, shares pathogenic features with CAD in other organs, such as the heart, kidney, and liver [4]. OB results from fibrotic processes triggered by T cells, antibodies, and innate immune cells in the allograft [5, 6]. Myofibroblasts, a class of mesenchymal cells that are predominantly transdifferentiated from fibroblasts or other mesenchymal cells, are the dominant collagen-producing cells in fibrotic diseases, including CAD [7–10]. Hence, suppressing the differentiation of myofibroblasts is a potential strategy for inhibiting CAD.

In addition to providing cellular energy, glycometabolism also regulates cell differentiation [11, 12]. As a member of the polo-like kinase (PLK) family, which mainly regulates mitosis and metabolic switching [13, 14], PLK1 regulates biosynthesis and metabolic switching by directly phosphorylating glucose-6-phosphate dehydrogenase (G6PD) and promoting the formation of its active dimer [15]. However, whether PLK1 has an effect on CAD remains unknown.

In this study, bioinformatic analyses revealed that the occurrence of CAD is accompanied by enhanced glucose metabolism, particularly via the pentose phosphate pathway (PPP). We subsequently tested target genes *in vivo*, and found that PLK1 is upregulated in rejected allografts compared to controls. We demonstrated that inhibiting PLK1 alleviates OB by suppressing the differentiation of myofibroblasts *in vivo*. Specifically, inhibiting PLK1 suppressed TGF-β1-mediated differentiation of myofibroblasts via non-Smad pathways. Thus, inhibiting myofibroblast differentiation by targeting PLK1 provides a potential new method to inhibit CAD.

## RESULTS

### The pentose phosphate pathway is enriched in dysfunctional allografts after lung transplantation

**Figure 1:**
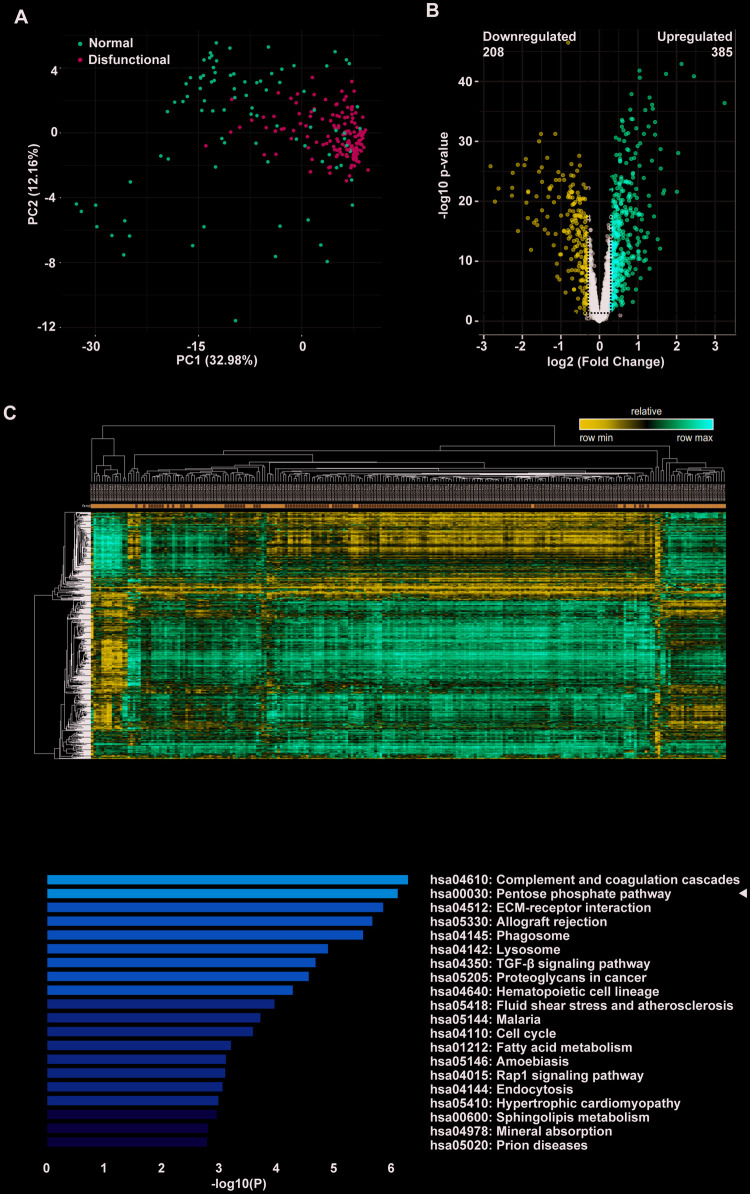
**The pentose phosphate pathway is enriched in tracheal transplantation allografts.** (**A**) Principal component analysis (PCA) plot of clinically-normal and -dysfunctional lung transplant biopsies in this dataset. The red dots represent the normal group while the green dots represent the dysfunctional group. (**B**) Volcano plot showing DEGs with p < 0.01 and fold change > 1.23; a total of 385 upregulated genes are represented by the red dots and 208 downregulated genes are represented by the blue dots. The black dots corresponds genes whose expression did not change. (**C**) Heatmap of lung transplant biopsies between the two indicated groups was obtained by unsupervised hierarchical clustering of DEGs and samples. Red indicates increased gene expression, whereas blue indicates decreased gene expression. (**D**) KEGG pathway enrichment of upregulated DEGs. The shades of the yellow bar correspond to -log10 (P), and the pentose phosphate pathway shows enrichment.

In a large sample study of consenting adult lung transplant recipients from seven centers, Kieran M Halloran and colleagues identified a set of rejection-associated transcripts. To identify pathways that might mediate allograft dysfunction after lung transplantation of patients, we performed DEG and functional enrichment analyses using public microarray data of a large number of clinical samples. DEGs were effectively divided into rejection and control groups as depicted in the PCA plot (Figure 1A). The volcano plot shows DEGs with p < 0.01 and fold change > 1.23; a total of 385 upregulated genes, and 208 downregulated genes (Figure 1B). A heatmap was obtained by unsupervised hierarchical clustering of the DEGs from the two lung transplant biopsy sample groups (Figure 1C). We performed functional analyses on the upregulated genes and found that a number of categories were particularly enriched. These included complement and coagulation cascades, allograft rejection, ECM-receptor interaction, and TGF-β signaling. As in several earlier studies, KEGG pathway enrichment analysis revealed that the PPP was significantly enriched (Figure 1D). As complement involved in antibody mediated rejection has been thoroughly researched, we focused on the PPP.

### The G6PD regulator PLK1 is highly expressed in murine tracheal allografts

**Figure 2:**
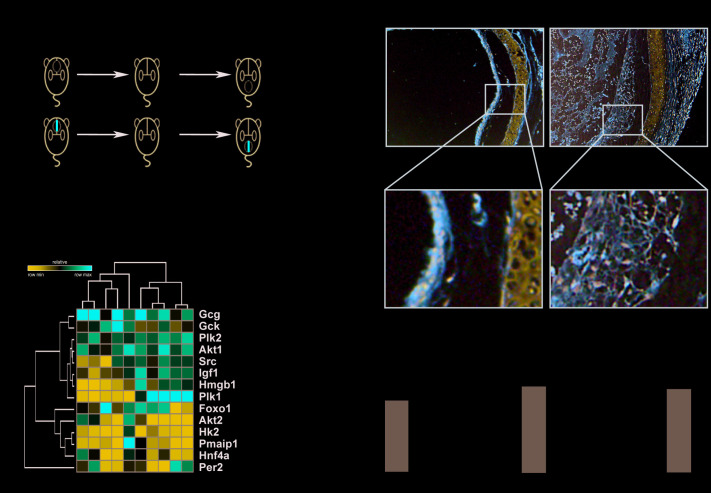
**The glucose-6-phosphate dehydrogenase (G6PD) regulator PLK1 is highly expressed in tracheal transplantation allografts.** (**A**) Schematic depicting the establishment of murine heterotopic trachea transplantation models. (**B**) Heatmap of relative expression values of 14 genes of interest from the microarray data validation as assessed by qRT-PCR in mouse. (**C**) Trachea grafts were harvested 28 days after transplantation, and the sections were stained for PLK1. (**D**, **E**) PLK1 expression in the allograft group compared with isograft group. (**F**) Total RNA was isolated from grafts and reverse-transcribed into cDNA. The transcript level of PLK1 relative to GAPDH was measured. * P≤0.05, ** P≤0.01, GAPDH; glyceraldehyde-3-phosphate dehydrogenase, PLK; polo-like kinase.

To ascertain the roles of glycolysis and the PPP in allograft rejection, we constructed a murine tracheal transplantation model. We obtained allografts 28 days after transplanting tracheas from BALB/c to C57 mice and used isografts of C57 to C57 mouse tracheal transplantations as controls (Figure 2A). We then analyzed the relative expression of key genes regulating glutaminolysis and the PPP. This revealed that, while HMGB1 and IGF1 were slightly upregulated [16, 17], PLK1 was the most upregulated gene (Figure 2B). IGF1 has been reported to contribute to recovery after liver transplantation, but the lack of a specific inhibitor makes it a suboptimal choice for treatment. Thus, we focused on PLK1 in subsequent experiments. Grafts were cut into sections and stained with PLK1 antibody (Figure 2C). We also detected PLK1 mRNA in homogenized tissue and calculated its relative mRNA levels by normalizing to GAPDH expression. This revealed that PLK1 was upregulated in allografts compared to isografts (fold change; 2.7±0.1, n=5, P<0.05, Figure 2D). The average optical density of PLK1 staining was also higher in allografts than in isografts (0.31±0.02 and 0.24±0.01, respectively, n=4, P<0.05, Figure 2E), as well as the percentage of positive staining area (0.23±0.02 and 0.17±0.01, respectively, n=4, P<0.05, Figure 2F).

### Inhibiting PLK1 can alleviate rejection in the early inflammatory phase

**Figure 3:**
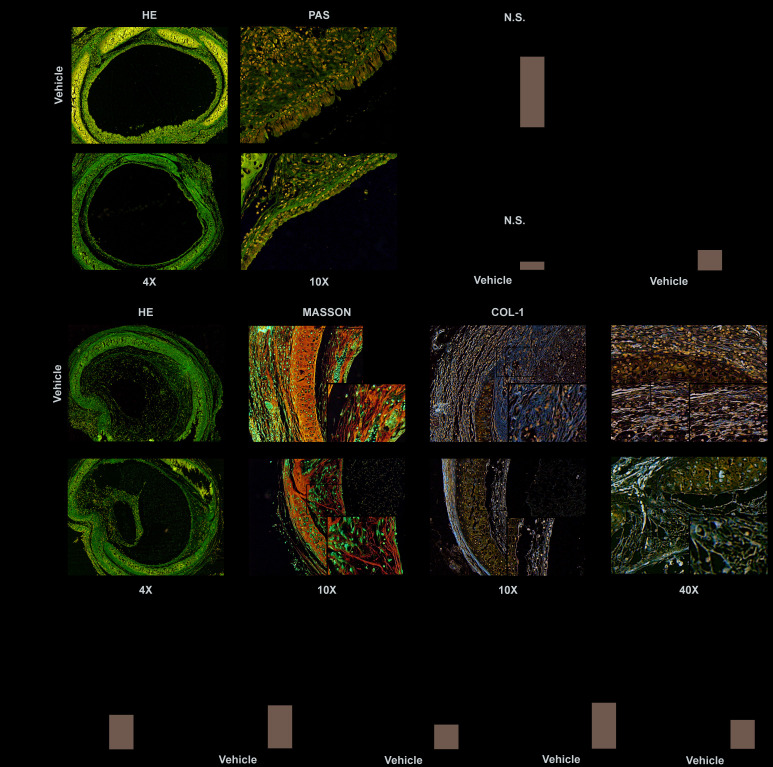
**PLK1 inhibition can alleviate rejection in the early and late inflammatory phases.** (**A**–**D**) BI6727 treatment alleviates airway epithelial loss 7 days after transplantation. (**A**) Representative H&E and PAS stains of tracheal grafts 7 days post-transplantation. (**B**) Percentage of luminal occlusion in the BI6727 treatment and control groups. (**C**, **D**) Sections were graded by two investigators independently, followed by blind treatment. (**E**) Representative immunohistochemical stains of tracheal grafts 28 days post-transplantation. (**F**) Percentage of luminal occlusion in the BI6727 treatment and control groups 28 days after transplantation. (**G**) Sections were graded by two investigators independently, followed by blind treatment. (**H**, **I**) The expression of collagenous fiber (**H**) and collagen I (**I**) in the BI6727 treatment and control groups 28 days after transplantation. (**J**) The percentage of α-SMA positive cells in the BI6727 treatment and control groups. * P≤0.05, H&E; haematoxylin-eosin, PLK; polo-like kinase, SMA; smooth muscle actin.

To evaluate the effect of PLK1 inhibition on the early phase of allograft rejection, we constructed a heterotopic tracheal transplantation model. Mice were treated twice a week by gavage with 25 mg/kg BI6727 (PLK1 inhibitor) dissolved in DMSO and corn oil, or DMSO and corn oil only (control group). We obtained allografts seven days after the operation. Grafts were cut into sections and stained with H&E and PAS (Figure 3A). There was no difference in the percentage of luminal occlusion between the BI6727-treated and control groups (19±1 and 22±1, P=0.09, n=5, Figure 3B). In addition, there was no difference in the grade of luminal obliteration between the BI6727-treated and control groups (0.4±0.2 and 0.5±0.2, n=10, Figure 3C). However, the grade of airway epithelial loss was reduced in the BI6727-treated group compared to that in the control group (1.0±0.3 and 2.8±0.4, respectively, n=10, P<0.05, Figure 3D).

### Inhibiting PLK1 can alleviate rejection in the late inflammatory phase

We next evaluated the effect of PLK1 inhibition on the later phase of allograft rejection by obtaining allografts 28 days after the operation. Grafts were cut into sections and stained by representative immunohistochemical dyes (Figure 3E). The percentage of luminal occlusion was reduced in the BI6727-treated group compared to that in the control group (32±3 and 84±3, respectively, n=6, Figure 3F), in accordance with the grade (2.0±0.3 and 3.8±0.3, respectively, n=8, Figure 3G). The expression of collagenous fiber was also reduced in the BI6727-treated group compared to that in the control group (0.07±0.01 and 0.23±0.01, respectively, n=6, Figure 3H), and the positive area was smaller in the BI6727-treated group than in the control group (0.21±0.02 and 0.35±0.01, respectively, n=6, P<0.05, Figure 3I). To distinguish myofibroblasts, we stained the sections with α-SMA antibody, and found a lower number of α-SMA+ cells in the BI6727 group than in the control group (0.14±0.02 and 0.37±0.03, respectively, n=6, P<0.05, Figure 3J).

### Inhibiting PLK1 suppresses TGF-β mediated myofibroblast differentiation

**Figure 4:**
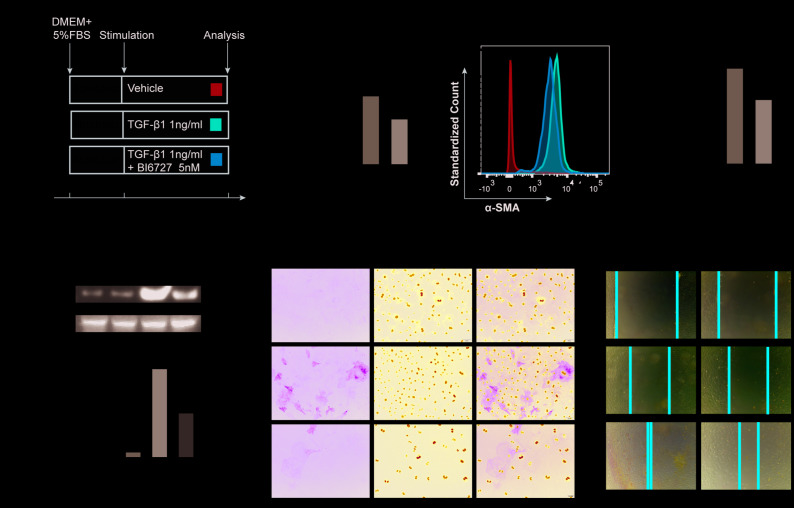
**PLK1 inhibition suppresses TGFβ-mediated differentiation of myofibroblasts.** (**A**) Schematic depicting establishment of the *in vitro* model of TGFβ-mediated differentiation of myofibroblasts. (**B**) Total RNA was isolated and reverse transcribed into cDNA. The transcript level of α-SMA in the BI6727 treatment group relative to the vehicle group was measured. (**C**, **D**) The expression of α-SMA was measured by FCM. Bar diagram shows MFI analysis of α-SMA expression. (**E**) The expression of α-SMA was measured by WB and quantified by densitometry. (**F**) The expression of α-SMA was analyzed by IF. (**G**) Migration ability of myofibroblasts in the vehicle and BI6727 treatment groups. * P≤0.05; FCM: flow cytometry; IF: immunofluorescence; SMA: smooth muscle actin; WB: western blot.

To further investigate the effect of PLK1 on myofibroblast differentiation, we used TGF-β1, the factor most commonly used for inducing differentiation of myofibroblasts (Figure 4A). Fibroblasts were first isolated from mouse embryos and purified by differential attachment. The cells were then treated with different concentrations of TGF-β1 and BI6727, and appropriate concentrations were chosen for use in subsequent experiments (Supplementary Figures 1, 2). We found that TGF-β1 did induce myofibroblast differentiation, as evidenced by increased expression of α-SMA, but this effect was reduced by BI6727 (Figure 4B–4F). Following isolation of total mRNA from the cells, we found that the transcriptional level of α-SMA was lower in the BI6727-treated group compared to the DMSO group (3.3±0.3, n=5 and 4.9±0.5, n=6, respectively, Figure 4B). Meanwhile, the expression of α-SMA protein decreased in BI6727-treated myofibroblasts compared to the control group, as assessed by FCM, WB, and IF (Figure 4C–4F). In addition, we tested the migration ability of the myofibroblasts, and found that BI6727 treatment had an inhibitory effect (Figure 4G).

### Transcriptomic profiling reveals that PLK1 inhibition affects TGF-β1-mediated gene expression in mouse myofibroblasts via the MAPK pathway

**Figure 5:**
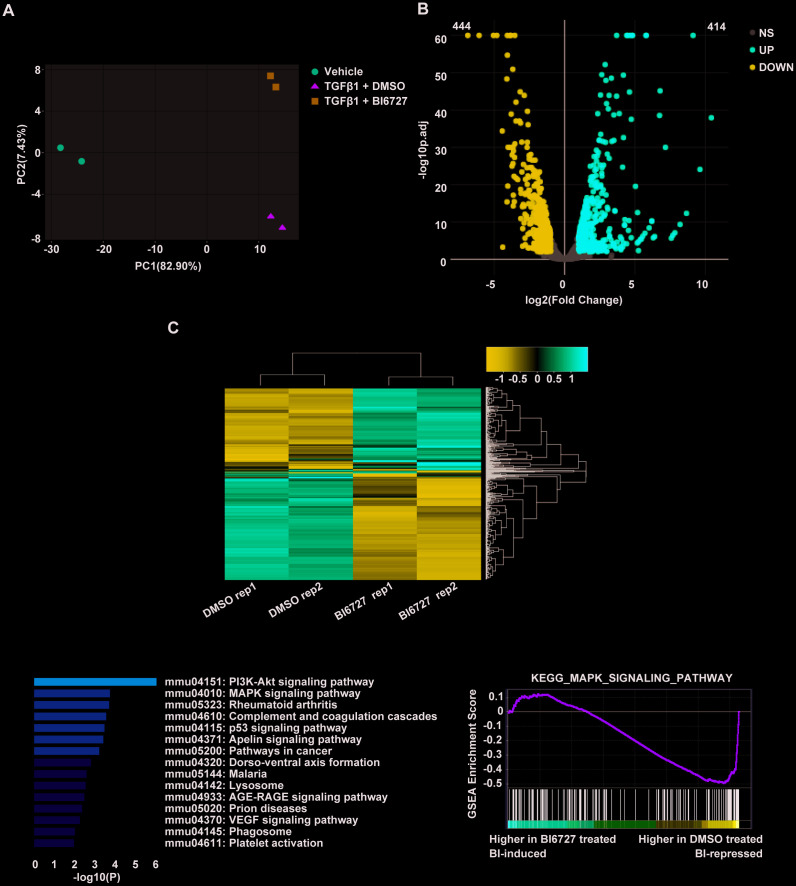
**Transcriptomic profiling reveals that PLK1 inhibition reduces myofibroblast differentiation in the late inflammatory phase via the MAPK pathway.** (**A**) Principal component analysis of six samples in the dataset. Each color represents one sample group. The red dot represents the vehicle group, the green triangle the TGF-β1+ DMSO group, and the blue square the TGF-β1+ BI6727 group. (**B**) Volcano plot of strongly upregulated (red; fold change > 2 and adjusted P value < 0.01) and downregulated (blue) genes in NIH-3T3 cells stimulated by TGF-β1 (1 ng/ml) vs DMSO (0.1%) for 72 h. There was a total of 414 upregulated and 444 downregulated genes. (**C**) Heatmap of differentially-expressed genes with BI6727 intervention. Red indicates increased gene expression, whereas blue indicates decreased gene expression. (**D**) KEGG pathway analysis of DEGs in PLK1-inhibited cells. The shades of the yellow bar correspond to -log10(P) Fisher’s exact test, used to select the significant (P < 0.05) pathways. (**E**) GSEA plot showing that PLK1 inhibition reduces myofibroblast differentiation in the late inflammatory phase via the MAPK pathway.

**Figure 6:**
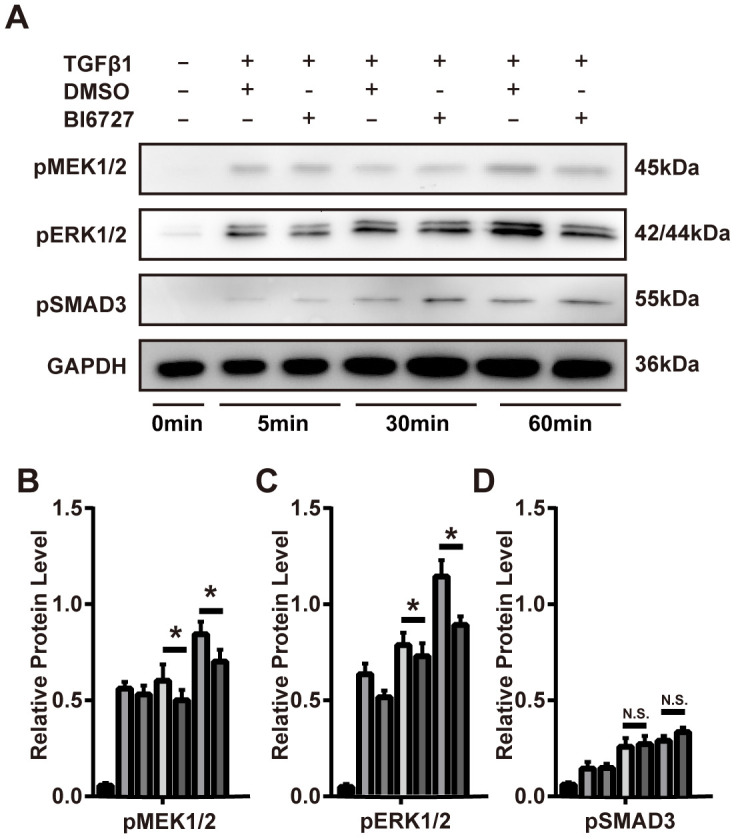
**PLK1 inhibition suppresses TGFβ-mediated differentiation of myofibroblasts via the MAPK-ERK pathway.** (**A**–**D**) The mouse myofibroblast TGF-β1 differentiation model was employed, with the addition of BI6727 to inhibit PLK1. Representative immunoblots for MEK, ERK, phosphorylated SMAD3, and GAPDH at various timepoints are shown. The results were quantified by densitometry, as represented in the bar diagrams. * P≤0.05.

To explore the cellular mechanisms underlying our findings in the mouse model of ectopic tracheal transplantation, we employed an *in vitro* cellular model of myofibroblast differentiation. NIH/3T3 cells were resuspended either in complete DMEM (group A, control), DMEM containing 1 ng/ml TGF-β1 and 0.1% DMSO (group B), or DMEM containing 1 ng/ml TGF-β1 and 5 μM BI6727 (group C), and cultured at 37 °C for 72 h. The cells were then washed with PBS and collected for extraction of total RNA. To assess the effect of PLK1 inhibition on the transcriptomes of myofibroblasts differentiating *in vitro,* two replicate RNA-seq experiments were performed for each condition. As depicted in the PCA plot, there was a high degree of consistency within each group (Figure 5A). We created the expression profile for 16713 genes. The volcano plot displays the DEGs between the TGF-β1-stimulated and control groups identified by applying the DESeq2 package, using P < 0.01 and fold change > 2 (414 upregulated genes, and 444 downregulated genes, Figure 5B). The corresponding heatmap is shown in Figure 5C. To confirm the relevance of these findings, we investigated the effects of PLK1 inhibition on myofibroblasts by pathway enrichment on the downregulated genes. This revealed various enriched categories, including complement and coagulation cascades [18, 19], platelet activation [20, 21], p53 signaling, lysosomes, AGE-RAGE signaling, VEGF signaling, phagosomes, and PI3K-Akt signaling (Figure 5D). Furthermore, we applied GSEA to the DEGs, which revealed that inhibiting PLK1 reduces myofibroblast differentiation in the late inflammatory phase in part via the MAPK pathway (Figure 5E). We further tested this potential molecular mechanism by western blotting, which revealed that the MAPK-ERK pathway was activated under TGF-β1 stimulation, whereas BI6727 treatment alleviated this change by reducing the phosphorylation of MEK and ERK (Figure 6A–6D).

### PLK1 siRNA treatment suppresses myofibroblast differentiation *in vitro* and *in vivo*

**Figure 7:**
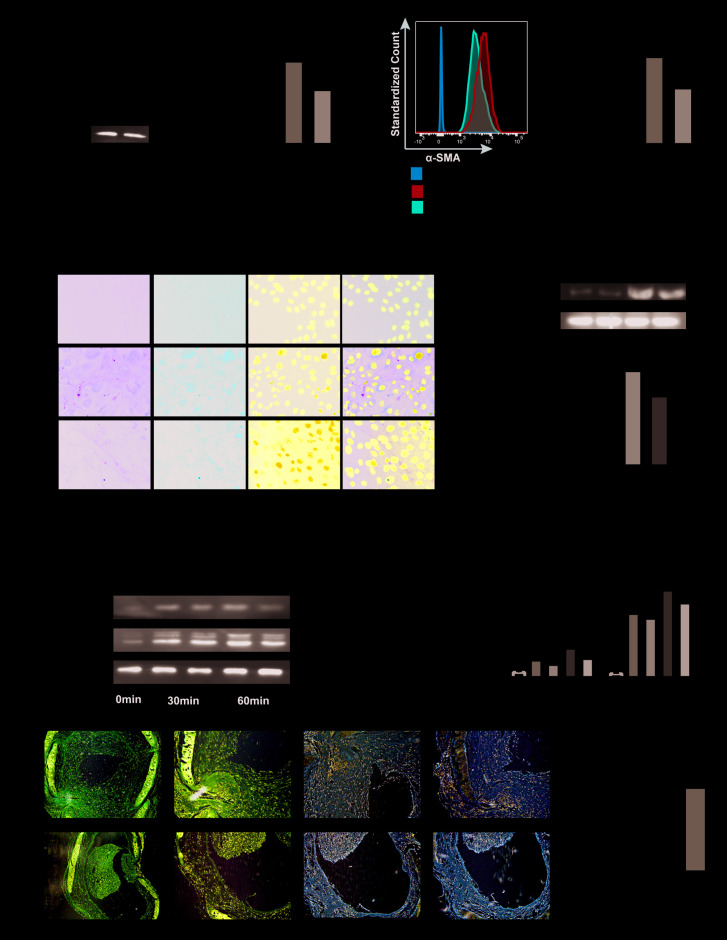
**PLK1 siRNA suppresses differentiation of myofibroblasts *in vitro* and *in vivo*.** (**A**) Protein expression of PLK1 following siRNA transfection. (**B**) The mRNA expression of α-SMA following PLK1 siRNA transfection. (**C**, **D**) The expression of α-SMA was measured by FCM; quantification by MFI analysis is shown in the bar diagram. (**E**) The expression of α-SMA was analyzed by IF. (**F**, **G**) The expression of α-SMA was analyzed by WB. (**H**, **I**) Representative immunoblots for MEK, phosphorylated ERK, and GAPDH at various timepoints are shown. (**J**) Representative immunohistochemical stains of PLK1 siRNA-treated or control tracheal grafts 28 days post-transplantation. (**K**) Percentage of luminal occlusion in the PLK1 siRNA-treated and control groups 28 days after transplantation.

To further verify the effect of PLK1 on the differentiation of myofibroblasts, we used siRNA to specifically knock down PLK1 *in vitro* and *in vivo*. We transfected siRNA into NIH/3T3 cells and confirmed their effectiveness in reducing PLK1 expression (Figure 7A). We also measured α-SMA expression at both the transcriptional and protein levels after 24 h PLK1 siRNA treatment, and obtained similar results as with BI6727 treatment (Figure 7B–7G). Phosphorylation of MEK and ERK was also inhibited by PLK1 siRNA treatment *in vitro* (Figure 7H, 7I). Next, we treated our heterotopic trachea transplantation models by intraperitoneally injecting PLK siRNA packaged with liposome. As observed with BI6727 treatment, PLK1 siRNA treatment alleviated rejection in the late inflammatory phase, 28 days after the operation (Figure 7J, 7K). Moreover, PLK1 siRNA treatment reduced the expression of α-SMA in the grafts. Overall, these results demonstrate that PLK1 siRNA treatment suppresses the differentiation of myofibroblasts *in vitro* and *in vivo*.

### PLK1 knockdown suppresses TGFβ-mediated differentiation of human myofibroblasts

**Figure 8:**
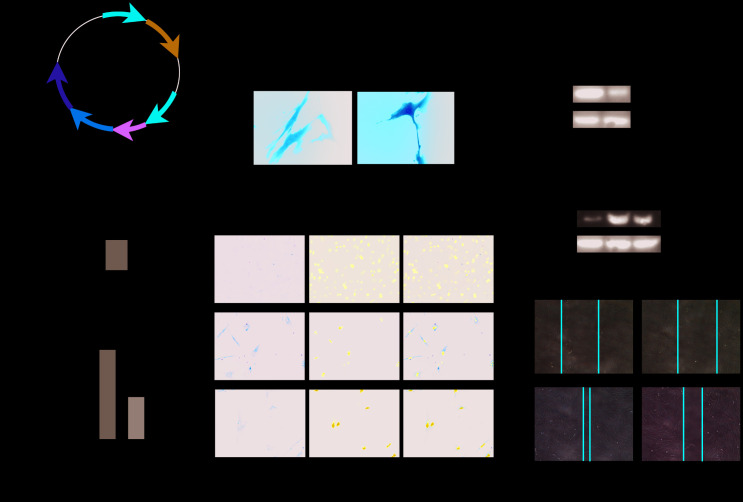
**PLK1 knockdown inhibits TGFβ-mediated differentiation of human myofibroblasts.** (**A**) Schematic depicting establishment of the recombinant plasmid RSV-PLK1-shRNA. (**B**) A representative image of infected human primary myofibroblast cells. (**C**–**F**) The expression of [50] α-SMA was analyzed by WB (**C**, **D**), qRT-PCR (**E**, **F**), and IF (**G**). (**H**) Migration ability of myofibroblasts in the control and PLK1-shRNA-treated groups.

Human primary fibroblasts were obtained as described in the Materials and Methods section above. We constructed the plasmid RSV-PLK1-shRNA and screened for cells with stable PLK1 knockdown using puromycin (Figure 8A, 8B). PLK1 knockdown decreased the expression of α-SMA (Figure 8C–8F) and the percentage of α-SMA+ myofibroblasts as detected by IF (Figure 8G). Similarly, the migration ability of the myofibroblasts was inhibited by PLK1 siRNA treatment (Figure 8H).

## DISCUSSION

During transplant rejection, metabolic reprogramming has been shown to occur in multiple cell types [22–24], particularly myofibroblasts, which are known to contribute to transplant-associated OB. Furthermore, TGF-β1 promotes myofibroblast differentiation via both Smad and non-Smad pathways [25, 26]. In our study, we describe a new therapeutic target to alleviate transplant-associated OB.

Glucose uptake and glycolysis are enhanced to meet increased needs for energy production; specifically, cells switch from fatty acid β-oxidation to aerobic glycolysis, and as a result depend more on glutaminolysis and the PPP [23, 24]. A previous study identified PLK1 as a master regulator of biosynthesis that accelerates cell proliferation and alters metabolic switching through activation of the PPP [15]. PLK1 increases PPP flux, NADPH, and ribose production for the synthesis of macromolecules, mainly by directly phosphorylating G6PD and promoting the formation of its active dimer [15, 27, 28].

Following *in vivo* verification of rejection-associated target genes, bioinformatic analysis led us to choose PLK1 as the major intervention target for subsequent experiments. Similar to previous studies, we observed myofibroblast accumulation in allotransplants, with a mass of extracellular matrix (ECM). Notably, allotransplants subjected to PLK1 inhibition had less myofibroblast accumulation and ECM than those treated with the vehicle control *in vivo* [29]. Murine myofibroblast differentiation induced by TGF-β1 was also suppressed by PLK1 inhibition *in vitro*. Importantly, we observed the same phenomenon in human primary fibroblasts.

Our study demonstrates that blocking PLK1 activity inhibits the differentiation of myofibroblasts. A primary feature of CAD is the occurrence of fibrosis in the allotransplant and its vessels [5]. Although studies have confirmed the involvement of many types of immune cells in this process, the use of immunosuppressor drugs has little effect on CAD [30, 31]. However, the specific function of myofibroblasts during chronic rejection after solid organ transplantation has now been recognized [7]. Myofibroblasts differentiate from fibroblasts, which are in turn mainly transdifferentiated from epithelial- or bone marrow-derived monocytes [9]. Studies exploring the function of myofibroblasts in CAD showed that blocking myofibroblast differentiation can effectively alleviate chronic rejection [25].

In this study, we made the surprising discovery that the G6PD regulator PLK1 was upregulated in the transplant rejection group. We therefore hypothesized that PLK1 promotes allograft rejection. Indeed, we proved that inhibiting PLK1 reduces transplant rejection both *in vitro* and *in vivo*. Similar to other reports, we demonstrated that PLK1 inhibition suppresses the MAPK-ERK pathway activated by the TGF-β type I receptor, whereas Smad3 was less affected by PLK1 inhibition [32]. Importantly, we also showed that the PLK1- TGF-β-MAPK-ERK pathway was active in human primary fibroblasts. This suggests that PLK1 could be a new therapeutic target to alleviate transplant-associated OB.

We also found that PLK1 inhibition suppresses inflammation during the early inflammatory phase (days 3-14). Interestingly, we also demonstrated that inhibiting PLK1 reduced the migration of fibroblasts, which, according to the “out-side-in” hypothesis, mainly migrate from peripheral blood [33]. We therefore speculate that PLK1 inhibits inflammation by inhibiting the migration of immune cells. PLK1 is a key molecule in glucose metabolism, and metabolism links many physiological processes, including migration and transdifferentiation [15, 34]. Our future studies will focus on the process of metabolic switching during chronic rejection, and on inhibiting this process by targeting glucose metabolism. Metabolomics is the quantitative analysis of all metabolite products in organisms. Analyzing the metabolome offers the potential to reveal the connection between glucose metabolism and CAD, potentially uncovering new biomarkers.

This study has several limitations. Although the heterotopic tracheal transplantation model has unique advantages in terms of recapitulating the accumulation of extracellular matrix (EMC) and the process of chronic rejection, it lacks normal hemoperfusion and ventilation to the external environment. Therefore, damage to the tracheal mucosa caused by pathogenic microorganisms and mechanical injury would not be taken into account using this model. Furthermore, PLK1 levels may have effects on cytokine expression and other immunological processes. Although models cannot perfectly simulate solid allotransplants *in vivo*, they suffice to prove that inhibiting PLK1 can suppress fibrosis. Future clinical studies are needed to test whether this phenomenon translates to useful clinical outcomes for lung transplant patients.

In conclusion, we have shown that inhibiting PLK1 can alleviate OB by suppressing TGF-β1-mediated differentiation of myofibroblasts via the MAPK-ERK pathways. For transplant patients with cancer, using checkpoint inhibitors or other immunoregulatory therapies can be difficult to balance, and our results provide a potential solution for this challenge [35–37]. PLK1 may represent a new target to prevent chronic transplant rejection. Further transplantation models and pathways should be tested to gain a deeper understanding of the mechanisms underlying BI6727’s (Volasertib) functions before trialing this drug in transplant patients.

## MATERIALS AND METHODS

### Animals, cells, and human participants

C57BL/6 (B6, H-2b) and BALB/c (H-2d) mice were maintained in Tongji Medical College, Huazhong University of Science and Technology (Wuhan, China), and 6 to 8 week-old male mice were used in our experiments. NIH/3T3 cells were authenticated using the STR (short tandem repeat) test (Boster, Wuhan, China). Human primary cells were collected from healthy volunteers who experienced resection operations. All patients involved in the study gave written, informed consent.

### Mouse model

Tracheal transplantation was performed as previously reported [38]. Briefly, tracheas were obtained from BALB/C mice and stitched onto the omentum majus of C57BL/6 mice. The experimental group was given 25 mg/kg BI6727 dissolved in corn oil by oral gavage twice a week, and the control group was given an equal volume of corn oil. GP-siRNA-Mate plus (GenePharma G04002, 1 ml) was used to transfect 0.75 OD siRNA by intraperitoneal injection once a week.

### Immunohistochemistry

Grafts were fixed in tissue fixing fluid (Servicebio, G1101) for 24 h, then paraffin-embedded and cut into sections for immunohistochemistry analysis. The sections were stained with haematoxylin and eosin (H&E) or another dyestuff [39]. Images were captured by microscopy and analyzed by Image-Pro Plus version 6.0 after background balancing (Media Cybernetics). The histological score was evaluated using recognized pathological criteria [40].

### Quantitative real-time PCR and siRNA transfection

Total RNA was extracted from grafts using Trizol reagent (Invitrogen, Carlsbad, CA) according to the manufacturer’s instructions, and cDNA was obtained using ABScript II RT Master Mix (Abclonal, RK20403). Real-time PCR was performed on a CFX Connect Real-time PCR System (BIO-RAD, California, USA) with 2X Universal SYBR Green Fast qPCR Mix (Abclonal, RK21203). The results were normalized to glyceraldehyde-phosphate dehydrogenase (GAPDH) gene expression. Primer sequences can be found in PrimerBank (https://pga.mgh.harvard.edu/primerbank/index.html), and siRNA sequences have been described previously [41]. All RNA was synthesized by Sangon Biotech (Shanghai), and siRNA transfections were performed as described previously [42].

### Flow cytometry

Cells were digested in a 0.05% pancreatic enzyme solution (Thermo Fisher, 25300054) for 3 min, and neutralized with Dulbecco's modified Eagle’s medium (DMEM, Boster, PYG0073) containing 10% fetal bovine serum (FBS). Cells were stained with Zombie Fixable Viability (BioLegend, San Diego, USA) before fixation by Fixation/Permeabilization Diluent (Thermofisher, 00-5223-56). Cells were stained with FITC Anti-alpha smooth muscle Actin (Abcam, ab8211, 1:50 dilution).

### Western blot analysis

Tissue or cells were lysed on ice using RIPA Lysis Buffer (Beyotime, P0013) containing Protease and Phosphatase Inhibitor Cocktail (Beyotime, P1050) for 30 min. Protein concentration is determined with the help of BSA standards (Beijing Solarbio Science & Technology Co., Ltd.). Western blotting was performed as previously described [43]. Anti-phospho-Smad3 (Beyotime, AF1759), anti- phospho-ERK1/2 (Cell Signaling Technology, 4370), anti-phospho-MEK1/2 (Cell Signaling Technology, 2338S), anti-PLK1 (Proteintech, 10305-1-AP), and anti-GAPDH (Proteintech, 60004-1-Ig) were used as primary antibodies. The intensity of bands was quantified using ImageJ software.

### Cell culture and immunofluorescence

NIH/3T3 or primary cells were starved in DMEM without FBS for 24 h, and then cultured in DMEM with 5% FBS and 5 nM hTGF-β1 (Peprotech, 500-M66) or 10 nM BI6727 (Selleck, S2235) for three days. Immunofluorescence was performed as previously described [44].

### Retrovirus-based plasmid preparation, virus production, and infection

For retroviral expression of human PLK1 (gene ID: 5347), we used pRSI9-U6-(sh)-UbiC-TagRFP-2A-Puro (Plasmid #28289; Addgene, MA, USA). Retrovirus-based shRNA was prepared by EZ-10 Column DNA Purification Kit (Sangon Biotech) and retroviral particles were produced according to a previous method [45, 46]. Fibroblast transduction was performed by incubating with the supernatant for 48 h. Puromycin was used to screen for expressing cells.

### RNA-seq

For *in vitro* cell experiments, NIH-3T3 cells were resuspended in complete DMEM (group A, control), or with the addition of either 1 ng/ml TGF-β1 (group B) or 1 ng/ml TGF-β1 and 5 μM BI6727 (group C) and cultured at 37 °C for 72 h, with two replicates in each group. After washing with PBS, cells were collected for extraction of total RNA, and three biological replicates of each of the two groups of cells were prepared. RNA was isolated using RNAprep pure Cell/Bacteria Kit (TIANGEN) according to the manufacturer’s instructions. The cDNA fragments with adapters were amplified by PCR, and the products were purified by Ampure XP Beads. Library quality was checked using an Agilent Technologies 2100 Bioanalyzer. The final library for RNA-seq was amplified with phi29 (Thermo Fisher Scientific, MA, USA) according to the protocol, and sequenced using a BGISEQ-500 sequencer (BGI) using single-end 50-cycle reads. The accession number assigned in the GEO database is GSE142732.

### RNA-seq analysis

The quality of the sequences was assessed using FASTQC v0.11.8 software in our bioinformatics server with Ubuntu 18.04.2 LTS. RNA-seq samples were analyzed using the Subjunc-FeatureCounts-DESeq2 pipeline. Clean reads were mapped to the mm10 (UCSC) mouse genome with Subjunc (v1.6.4), using the default parameters. Expression quantification of transcripts was performed using FeatureCounts (v1.6.4) [47] with mouse annotation (release_M20) from Gencode [48]. Genes with at least 10 reads per million mapped reads in more than three samples were considered to be expressed. Differential gene expression was analyzed using the DESeq2 package (v1.22.2) [49] of the R software (version 3.6.1) [50]. Genes with less than 5% probability of being false positives (p-adjusted < 0.01) and an absolute fold change > 1 were chosen for subsequent downstream functional analyses. The volcano plot, PCA, and heatmap data were plotted in DEBrowser [48].

### Functional annotation of differentially expressed genes (DEGs)

Functional annotation of Gene Ontology or Kyoto Encyclopedia of Genes and Genomes (KEGG) pathway enrichment analysis for sets of differentially expressed genes (DEGs) were performed using metascape (http://metascape.org). The thresholds were p < 0.05 and FDR < 0.05.

### Gene set enrichment analysis

Gene set enrichment analysis was performed by GSEA (v3.0) [51, 52] software downloaded from the Broad Institute (http://software.broadinstitute.org/gsea). Statistical analysis for RNA-seq are described in detail above. GSEA was performed as previously described [52]. Enrichment score curves and member ranks were generated by the GSEA software (Broad Institute).

### Statistical analysis

Data are presented as mean ± SD. Comparisons between the data were calculated using a two-tailed Student’s *t*-test. All the data were analyzed using Prism 7.0. We considered *P < 0.05 as statistically significant.

## Supplementary Material

Supplementary Figures
