# Supplementary material for: PLK1 Inhibition alleviates transplant-associated obliterative bronchiolitis by suppressing myofibroblast differentiation
Source: Aging (Albany NY). 2020 Jun 15;12(12):11636–52. doi: 10.18632/aging.103330 (PMC7343459; doi:10.18632/aging.103330)
Supplement: Supplementary Figures [file aging-12-103330-s001..pdf]

SUPPLEMENTARY FIGURES

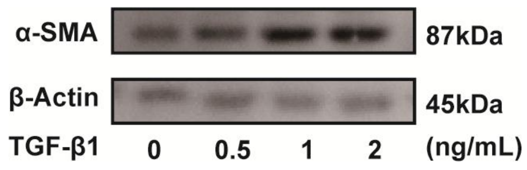

Supplementary Figure 1. Fibroblasts were stimulated by different concentrations of TGF-β1.

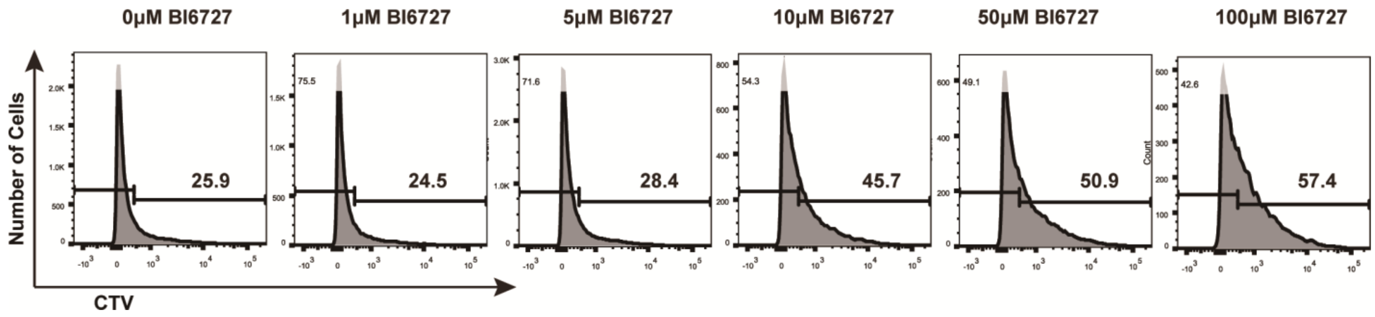

Supplementary Figure 2. Fibroblast proliferation is not affected by BI6727 treatment for 72 hours.
